# Supplementary material for: Minimising efficiency roll-off in high-brightness perovskite light-emitting diodes
Source: Nat Commun. 2018 Feb 9;9:608. doi: 10.1038/s41467-018-03049-7 (PMC5807308; doi:10.1038/s41467-018-03049-7)
Supplement: Supplementary file 1 — Supplementary Information [file 41467_2018_3049_MOESM1_ESM.pdf]

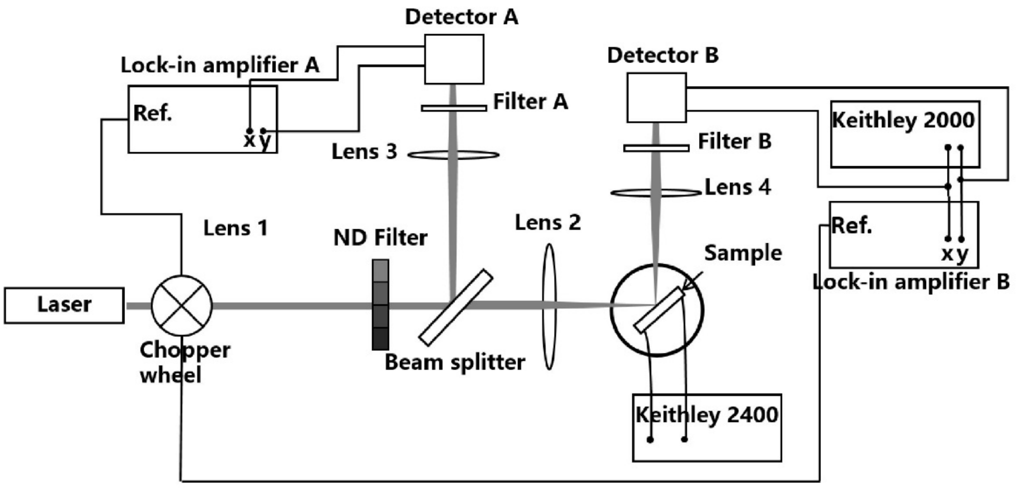


**Supplementary Figure 1. Layout of the PL and EQE measurement setup.** The PL and EQE of the devices were measured simultaneously by using a chopped laser when the LED device is under operation.

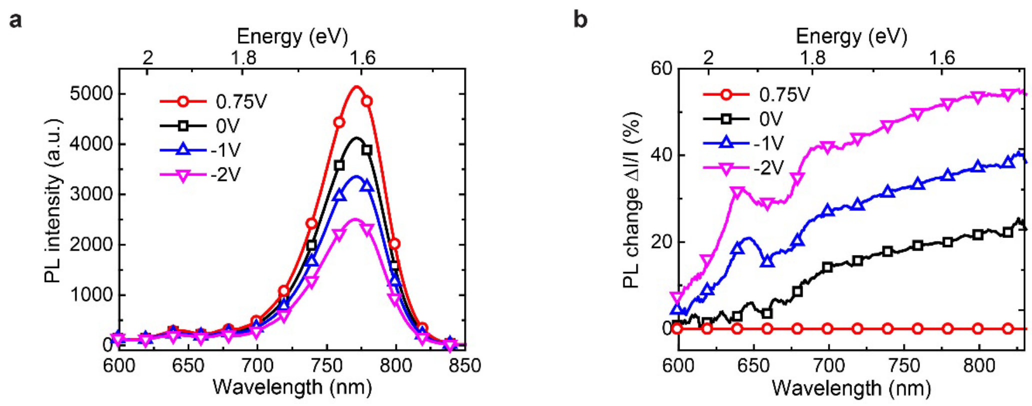


**Supplementary Figure 2. PL of MQW perovskite LEDs.** (**a**) PL spectra of the device under different driving voltages. (**b**) Change of PL intensity at selected driving voltages, relative to the PL intensity at 0.75 V ((PL(0.75 V)-PL(V))/PL(0.75 V)).

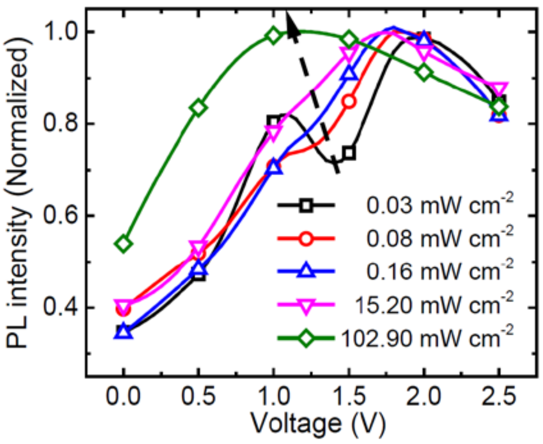


**Supplementary Figure 3. Bias-dependent PL intensities (normalized) under various photo-excitation intensities.** With increasing excitation intensities, the effect of PL quenching decreases and the trough stemmed from the competition between field-induced quenching and exciton screening gradually disappears.

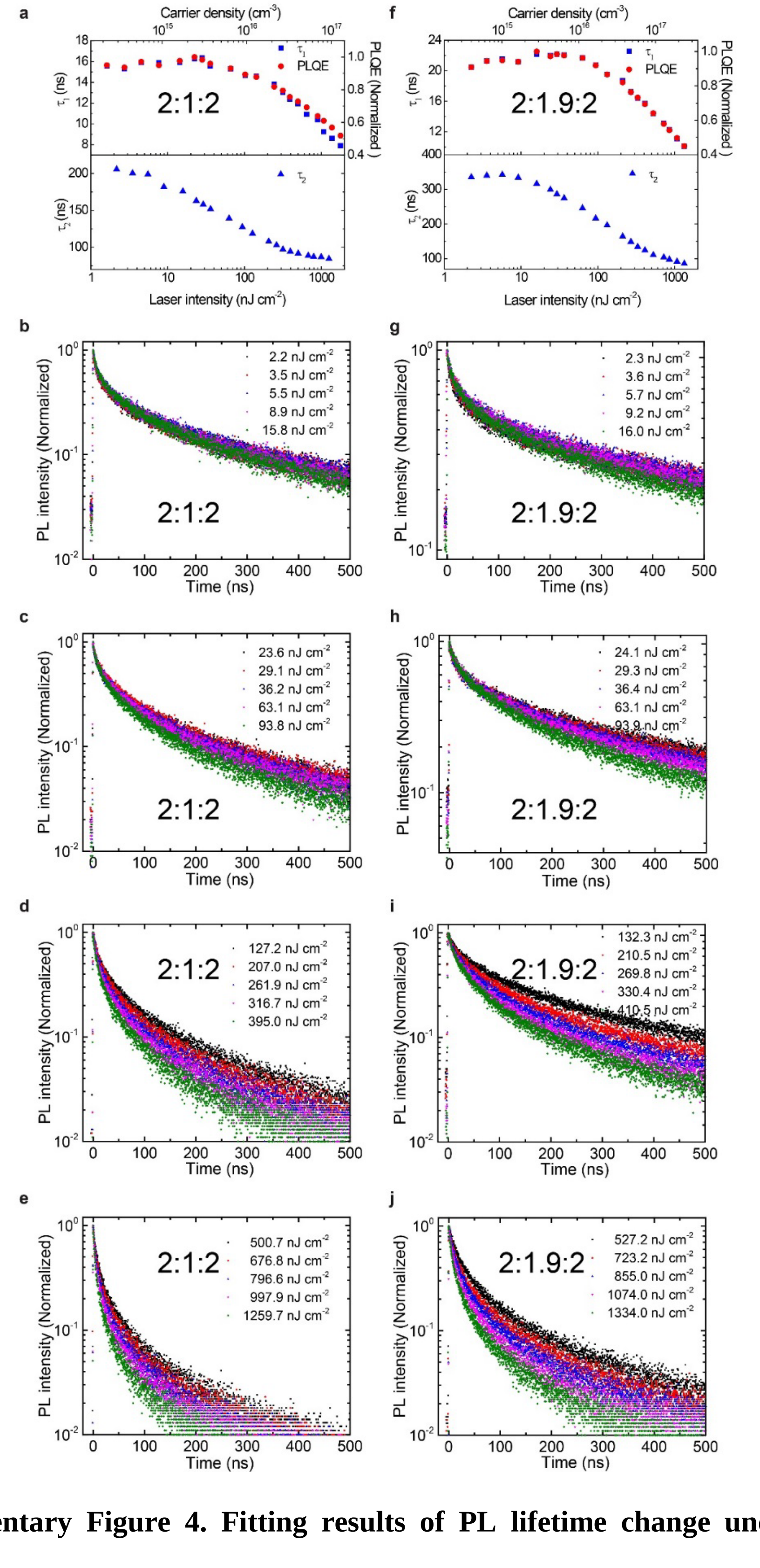


**Supplementary Figure 4. Fitting results of PL lifetime change under various excitation intensities.** Excitation intensity-dependent PL lifetimes of the (**a)** 2:1:2 and (**f**) 2:1.9:2 $NFPI_7$ MQW films. PL decay traces of the (**b**,**c**,**d**,**e**) 2:1:2 and (**g**,**h**,**i**,**j**) 2:1.9:2 $NFPI_7$ MQW films under different excitation intensities.

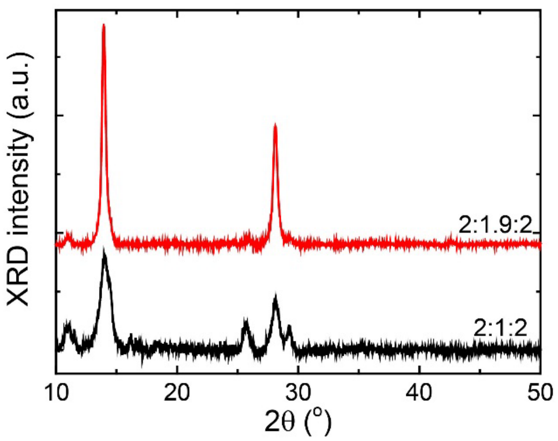


**Supplementary Figure 5. XRD data of the 2:1:2 and 2:1.9:2 perovskite MQW films.** The MQW films consist of small-n layered QWs and large-n QWs[1,2]. The peaks at 13.9° and 28.1° are consistent with the diffraction peaks from (111) and (222) crystal planes of 3D $FAPbI_3$.

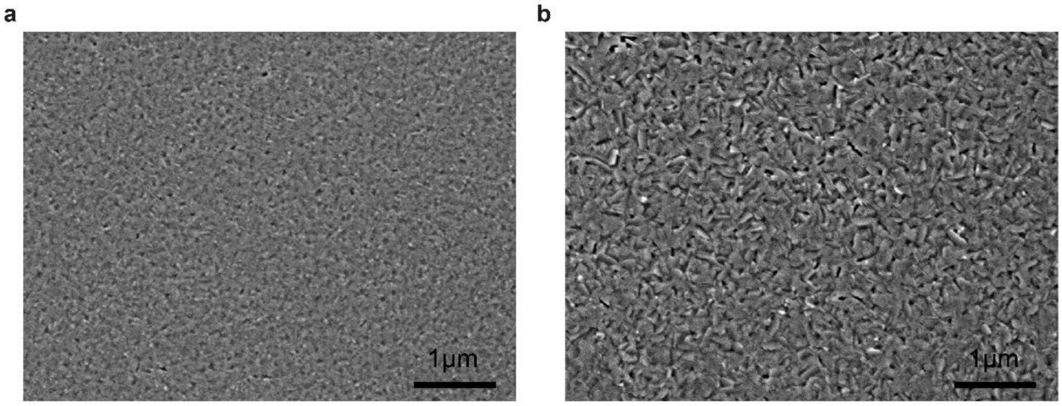


**Supplementary Figure 6. SEM images of the perovskite MQW films. (a**) 2:1:2. (**b**) 2:1.9:2. Scale bar: 1 μm.

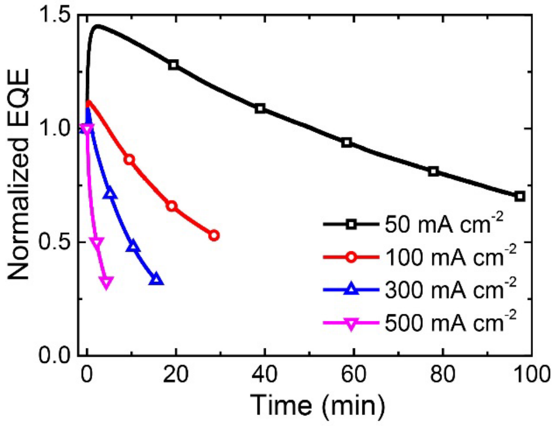


**Supplementary Figure 7. Stability data of the perovskite MQW LEDs at different constant current densities.**

## Supplementary References

1. Quan, L. N. *et al.* Ligand-stabilized reduced-dimensionality perovskites. *J. Am. Chem. Soc.* **138,** 2649–2655 (2016).

2. Pang, S. *et al.* $NH_2CH{=}NH_2PbI_3$: An alternative organolead iodide perovskite sensitizer for mesoscopic solar cells. *Chem. Mater.* **26,** 1485–1491 (2014).
